# Tip of the clade on the top of the World—the first fossil Lophopidae (Hemiptera: Fulgoromorpha) from the Palaeocene of Tibet

**DOI:** 10.1007/s00114-015-1277-4

**Published:** 2015-04-28

**Authors:** Jacek Szwedo, Adam Stroiński, Qibin Lin

**Affiliations:** Department of Zoology and Parasitology, University of Gdańsk, 59, Wita Stwosza St., PL80-308 Gdańsk, Poland; Museum and Institute of Zoology, Polish Academy of Sciences, 64, Wilcza Street, PL00-679 Warszawa, Poland; State Key Laboratory of Palaeobiology and Stratigraphy, Nanjing Institute of Geology and Palaeontology, Chinese Academy of Sciences, Nanjing, 210008 People’s Republic of China

**Keywords:** Insecta, Lophopidae, Palaeocene, Tibet, Phylogeny, Biogeography, Taxonomy

## Abstract

Lophopidae is a family of planthoppers (Hemiptera: Fulgoromorpha) present today in tropical and subtropical zones of the Old World. The most recent taxonomic studies and phylogeny of these insects do not include the extinct representatives. Therefore, each new discovery of a fossil lophopid is of high interest, giving new insights to their evolutionary history and enabling to test the proposed relationships. The recent findings of extinct Lophopidae in Europe, in various Palaeogene deposits, put in doubts their proposed evolutionary and biogeographic scenario. The new fossil from the Palaeocene of Northern Tibet is related to one of the Lophopidae clades, Apia^+^ group, believed to be the most advanced one, and recently distributed in the recent Sundaland-New Guinea-Queensland area. A new genus and species *Gesaris gnapo* gen. et sp. n. provide information on early lophopids diversity and relationships and demonstrates the necessity for a revision of the existing hypotheses for the initial diversification and distributional pattern of the Lophopidae.

**Electronic supplementary material:**

The online version of this article (doi:10.1007/s00114-015-1277-4) contains supplementary material, which is available to authorized users.

## Introduction

The planthopper family Lophopidae Stål, 1866 is one of the smallest within Fulgoroidea, with 43 genera and over 140 species recognized, both recent and extinct (Bourgoin 2015; Szwedo 2011; Stroiński and Szwedo 2012). This tropical Old World family (except *Carriona* Muir, 1931 from Peru, Ecuador and Panama) is the first to have a modern generic level phylogenetic analysis, biogeographic scenarios proposed and host plants relationships discussed (Soulier-Perkins 1998, 2000, 2001; Soulier-Perkins et al. 2007, 2013; Szwedo and Soulier-Perkins 2010). The members of the family could be identified by unique combination of characters of head, legs, and tegmina (Soulier-Perkins 1998; Soulier-Perkins et al. 2013). There are a few known fossils ascribed to Lophopidae (see Supplementary Material for a full list and Fig. 2) known since Palaeocene to late Eocene.

**Fig. 1:**
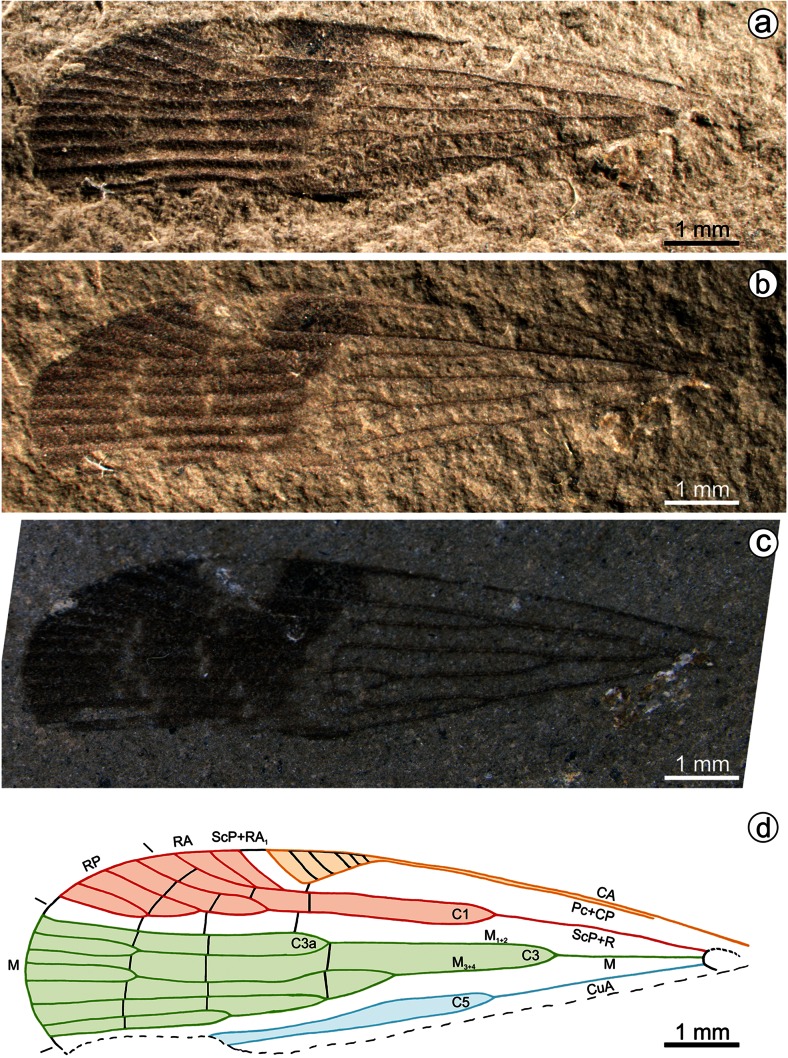
*Gesaris gnapo* gen. et sp. n. **a**–**c** Photographs of the specimen under different light conditions. **a**, **b** Dry specimen under light from sides, **c** specimen under alcohol. **d** Reconstruction of tegmen venation

**Fig. 2:**
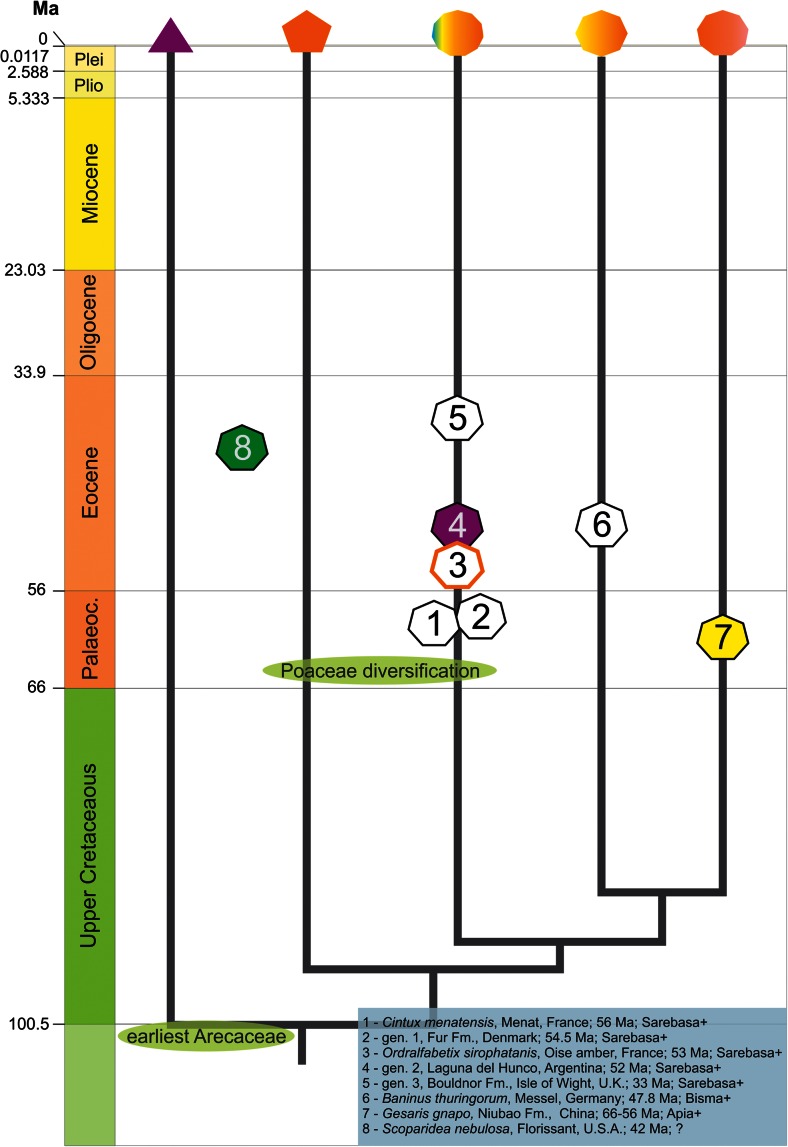
Time and space distribution of fossil Lophopidae on the phylogenetic tree of lineages (generic groups) as proposed by Soulier-Perkins (2000, 2001)

## Material and methods

The specimen was examined using dissecting stereoscopic microscope Nikon 1500 and illustrated with the aid of the drawing tube. Photographs were taken using Nikon Digital camera DXM and Zeiss SteREO Discovery.V20 system. The venation nomenclature follows Bourgoin et al. (2015). The generic groups are marked according to the convention proposed by Amorim (1982), i.e., the name given to a monophyletic group is the name of the more basal taxon in this, followed by the sign ^+^

## Systematic paleontology

### Family Lophopidae Stål, 1866

*Gesaris* gen. n.

#### Type species

*Gesaris gnapo* sp. n. by monotypy and present designation.

#### Diagnosis

Pattern of venation close to the recent genus *Maana* Soulier-Perkins 1998. Costal area narrow at base without transverse veinlets, apical portion distinctly widened with a few veinlets (similar pattern in *Maana*); three rows of veinlets—nodal, subapical, and apical one present (as in *Maana* and other genera of the Apia^+^ group); it differs from *Maana* and other genera of Apia^+^ group by the sequence of forking of main stems—M → ScP + R = CuA (model ScP + R → M → CuA in the other genera of the Apia^+^ group); branches M_3+4_ and CuA_1_ without common portion (branches M_3+4_ and CuA_1_ with a short common portion in *Maana* and other members of the Apia^+^ group present).

#### Etymology

Gesaris—name of the warrior king from the Tibetan, Chinese, and Mongolian mythology. Gender: masculine, third declension.

#### Composition

Only type species *Gesaris gnapo* sp. n.

#### Description

Tegmen narrow, about 3.5 times as long as wide, veins on membrane strongly elevated, carinate, with three rows of veinlets: nodal, subapical, and apical ones. Costal margin almost straight, costal area very narrow, parallel to costal margin, distinctly widened in the apical portion, forming ‘stigmal area’ (near nodal line); basal portion of costal area without veinlets, apical, widened portion (‘stigmal area’) with five oblique veinlets. Stems ScP + R, M, and CuA leaving basal cell separately. Stem ScP + R forked apicad of stem M forking, at same level as stem CuA forking. Branch ScP + RA forked slightly apicad of nodal line, with three terminals reaching margin. Branch RP forked apicad of nodal line, then forked subsequently at level of subapical and apical lines, reaching margin with four terminals. Stem M forked at basal $$ \raisebox{1ex}{$1$}\!\left/ \!\raisebox{-1ex}{$3$}\right. $$ of tegmen length, distinctly basad of stems ScP + R and CuA forkings. Stem M forked basad of stems ScP + R and CuA forkings. Branch M_1+2_ forked at level of nodal line, branch M_3+4_ forked distinctly basad of nodal line, branch M_4_ forked at nodal line. Stem CuA forked at same level as stem ScP + R forking. Nodal line composed of veinlets: *pccp*-*scpra* (between Pc + CP and ScP + R), *1ir*, *1r*-*m*, and two veinlets *1im*. Subapical line present was composed of veinlets *2ir*, *2r*-*m*, and *2im*. Apical line present was composed of veinlets *3ir*, *3r*-*m*, and *3im*.

Cell C1 narrower than postcostal cell, closed apically with nodal line veinlet *1ir*. Cell C3 longer and wider than cell C1, closed with nodal veinlet *1im*. Cell C3a present basad of nodal line. Cell C5 narrower than cells C1 and C5, elongate, lancet-shaped.

Postnodal cells about as long as apical cells, subapical cells shorter than apical cells.

#### Remarks

Based on the venational patterns, *Gesaris* gen. n. belongs to the Apia^+^ clade as delimited by Soulier-Perkins (2000, 2001). This generic group comprises nine extant genera: *Acarna* Stål, 1863; *Apia* Distant, 1909; *Jugoda* Melichar, 1915; *Kasserota* Distant, 1906; *Maana* Soulier-Perkins 1998; *Magia* Distant, 1907; *Megacarna* Baker, 1925; *Onycta* Fennah, 1955; and *Zophiuma* Fennah, 1955. Within this group, the subunit encircling *Maana*, *Kasserota*, *Acarna*, *Magia*, and *Onycta* form a separate subclade. This subclade could be delimited by the elongate tegmina with sparse but regular lines of veinlets on membrane: nodal, subapical, and apical ones; very narrow costal area, with veins CA and Pc + CP very close each other in basal portion, always without veinlets in between and widened in apical part, with veinlets in ‘stigmal area’.

*Gesaris gnapo* sp. n.

(Fig. 1a–d)

#### Diagnosis

Tegmen hyperpterous in RP and M (sensu Bourgoin et al. 2015). Stem ScP + RA reaching margin with three terminals: ScPRA_1_, RA_2_, and RA_3_; stem RP forking pectinate, reaching margin with four terminals. Stem M reaching margin with eight terminals. Cell C3a 0.35 × as long as cell C3.

#### Etymology

Specific epithet is derived from the Tibetan word ‘gna’ po’ meaning primeval, ancient.

#### Holotype

Specimen No. NIGP 135805. Imprint of tegmen with clavus missing and postclaval margin partly destroyed. Deposited in the collection of the Nanjing Institute of Geology and Palaeontology, Chinese Academy of Sciences, Nanjing, China.

#### Type locality, horizon, and age

Gangni Village, Anduo County, Dazhuoma area of the Qiangtang Basin in northern Tibet; Niubao Formation; Palaeocene.

#### Description

As for the genus. Length of tegmen 9.6 mm, width 2.72 mm. Cell C1 2.48 mm long, cell C3 3.32 mm long, cell C3a 1.16 mm long.

## Discussion

The ancestor of the family Lophopidae was postulated as feeding on Arecaceae (Fig. 2), with two later changes to Poaceae and Musaceae (Soulier-Perkins et al. 2007). Three scenarios were made to explain the paradoxical biogeographic distribution of the Lophopidae based on different geological events and times (Soulier-Perkins 2000). After a new assessment of the existing data on fossil and recent Lophopidae and their postulated host plants, any of them match to the observed facts. The oldest fossil species of Lophopidae are now from Tibet (*Gesaris gnapo* gen. sp. n.—Apia^+^ group) and Europe (*Cintux menatensis* Stroiński et Szwedo, 2012—Sarebasa^+^ group). Then, it could be assumed that Lophopidae has separated in Late Cretaceous, benefited from exploitation of the new habitats and host plants expansion due to Mid-Cretaceous re-organisation of biosphere (Szwedo and Soulier-Perkins 2010; Stroiński and Szwedo 2012). It seems probable that this separation took place somewhere in the ancestral area of Arecaceae and that Lophopidae committed a rather rapid diversification and spreading coincident with them. The ecological shift of Sarebasa^+^ clade to Poaceae was postulated to take place in South-East Asia (Soulier-Perkins et al. 2007). This shift could be related to Poaceae massive diversification end expansion in the Palaeogene (Jones et al. 2014; Magallón et al. 2015). Only a sole species of monotypic genus—*Megacarna albosparsa* (Melichar, 1913) shifted to Musaceae. Soulier-Perkins (2000) postulated that ancestors of the Bisma^+^ group (to which the Apia^+^ clade comprises) originated in the West Pacific islands arc. Eleven of the concerned genera are found in terranes originating from this arc. However, the oldest fossil ascribed to the Bisma^+^ group—*Baninus thuringiorum* Szwedo et Wappler, 2006—comes from the Middle Eocene Messel Maar (Szwedo and Wappler 2006). Finding of the representative of the Apia^+^ group, the group believed to be the most advanced among the Lophopidae in the Palaeocene of Qiangtang Basin pushes back the time of separation of at least of this group of genera, but also questions the postulated area of origination. Ancestors of the modern Apia^+^ group could originate earlier than formerly supposed (Soulier-Perkins 2000). The presence of extinct members of the Bisma^+^ group in Europe (Szwedo and Wappler 2006) could support this statement. The early diversification and westward migrations of ancient Lophopidae probably took place in the early Palaeocene, due to suitable palaeogeographic and climatic conditions (Martin et al. 2013). It could be assumed that the ‘invasion’ of lophopids to the Indian subcontinent resulted from docking of it to mainland Asia. The palaeogeographic situation of the area is very complicated, a number of competing models for the Cretaceous evolution of the Tethys ocean between India and Eurasia were proposed, and these need to be understood both in the context of deformation in SE Asia, as well as in the Himalayas (Hall 2012). Also climatic changes—the Eocene Thermal Maximum (ETM2) and the subsequent Early Eocene Climatic Optimum (Zachos et al. 2008) influenced this expansion. Following climatic and biotic events of late Palaeogene and Neogene (Shukla et al. 2013) left the isolated genus *Bisma* Distant, 1907 in Ceylon as a relic of wider distribution in the past. Ancestors of the recent genera of the Apia^+^ group could reach New Guinea at about 25–20 Mya, when the East Philippines − Jalmaher − South Caroline Arc collided with the Australian Plate at the north New Guinea margin (Hall et al. 2011). Ancestors of recent *Magia* species probably ‘invaded’ Australia later, during Pliocene-Pleistocene (Soulier-Perkins 2000). The recent genera placed in the Apia^+^ group seem to be relatively young descendants of the much older ancestral forms, present in the Palaeocene. Such evolutionary history of the Apia^+^ group seems to be reflected in its recent distribution (Soulier-Perkins 2000) and trophic relationships of the recent taxa (Soulier-Perkins 2007).

## Conclusion

It could be concluded that discovery of fossil Lophopidae in Palaeocene deposits of Tibet gives a new clue to evolutionary and distributional patterns of the Lophopidae. The recent distribution of this group and its subunits seems to be result of millions of years of dispersal and extinction events, as well as vicariance events in some areas, influenced by changes of the availability of host plants, host-plants shifting and biotic and climatic changes at global and local scale. Scarce data on fossil insects from the most crucial period of Palaeogene faunistic turnover in Asia after the collision with Indian plate are available (Lin et al. 2010; Szwedo et al. 2013). Fossils from Tibet can bring new insights not only to evolution of groups, but also into paleoevents of biotic reorganization and formation of modern fauna of Asia.

## Electronic supplementary material

ESM 1 (DOC 75 kb)
