# Supplementary material for: Tip of the clade on the top of the World—the first fossil Lophopidae (Hemiptera: Fulgoromorpha) from the Palaeocene of Tibet
Source: Naturwissenschaften. 2015 Apr 28;102(5-6):28. doi: 10.1007/s00114-015-1277-4 (PMC4412278; doi:10.1007/s00114-015-1277-4)
Supplement: Supplementary file 1 — (DOC 75 kb) [file 114_2015_1277_MOESM1_ESM.doc]

Supplementary material

**Fossil site**

A geological team from the Jianghan Institution of Petroleum and Gas collected a large number of fossils in the Qiangtang Basin of the Northern Tibet, China; insects collected near Gagni village were sent to one of us (QBL) for identification. Preliminary, 19 insect morphospecies attributed to 17 genera and 11 families are recorded from the Dazhuoma Entomofauna (near Gangni village), indicating a rather distinct diversity (Lin and Huang 2006). Only a single species of Prophalangopsidae (Orthoptera) and single species of Flatidae (Hemiptera) were described so far (Lin and Huang 2006; Szwedo et al. 2013). The fossil insects occur mainly in the lower part of the second member of the Niubao Formation (beds 179–249). The assemblage is diversified and quite abundant in individuals. The age of Dazhuoma Entomofauna was estimated as Palaeocene to Early Eocene in age (Cai and Fu 2003) and more recently aged Palaeocene (Lin and Huang 2006). Lithology of the fossil site was not reported and no more precise data are available (Zhang S 2009).

**Fossil Lophopidae**

Fossil Lophopidae are not very common and fossil record of the family comprises: *Cintux menatensis* Stroiński et Szwedo, 2012 from the Palaeocene of Menat in France, *Ordralfabetix sirophantes* Szwedo, 2011 from Lowermost Eocene Oise amber of France and *Baninus thuringiorum* Szwedo and Wappler, 2006 from the middle Eocene of Messel in Germany (Stroiński and Szwedo 2012; Szwedo 2011; Szwedo and Wappler 2006). Two more fossils of Lophopidae were reported by Petrulevičius et al. (2010a, b): one coming from the Upper Palaeocene Fur and Ølst Formations in Denmark, and the second from the Lower Eocene Laguna del Hunco, Argentina. The last European record of Lophopidae comes from the late Eocene deposits of Isle of Wight, U.K. (Szwedo, unpubl. data). The taxonomic placement of the only fossil lophopid from North America, i.e. *Scoparidea nebulosa* Cockerell, 1920 from the Eocene Green River Formation, Roan Mountain, Colorado, U.S.A. (Cockerell 1920) remains uncertain (Szwedo 2011), though it presents some salient features of the family.

**Arecaceae as ancestral host plants of Lophopidae**

The ancestor of the family Lophopidae was postulated as feeding on Arecaceae, with two later changes to Poaceae and Musaceae (Soulier-Perkins et al. 2007). According to Stevens (2015) and Magallón et al. (2015) all these families are placed in well supported monophyletic clade of monocotyledons (monocots) plants – commelinids. Arecaceae is a monophyletic group recently including 188 genera and approximately 2585 species (Govaerts and Dransfield 2005; Dransfield et al. 2008; Baker and Couvreur 2012, 2013a, b; PALMweb 2015). They are recently distributed in the tropics, with a few species reaching subtropical areas (Henderson et al. 1997, Eisenhardt et al. 2011). Their fossil records from the tropics are less common, however, than those from middle and high latitudes (Harley and Morley 1995; Harley and Baker 2001; Harley 2006; Palazessi and Barreda 2007; Dransfield et al. 2008; Gomez-Navarro et al. 2009; Futey et al. 2012). Fossil record of Arecaceae reflects their broader latitudinal distribution in the late Mesozoic and early Cenozoic because of warmer global climate (Zachos et al. 2008, Friedrich et al. 2012), but also lack of study in the early Cenozoic deposits in the tropics (Pan et al. 2006). The earliest unequivocal Arecaceae fossils are from the late Mid-Cretaceous to early Upper Cretaceous (Harley 2006; Dransfield et al. 2008; Couvreur et al. 2011, Futey et al. 2012). Australasia (including New Guinea, New Caledonia, and New Zealand) was hypothesized as ancestral area of Arecaceae and their crown node is estimated to be ca. 110 Myr old (Janssen and Bremer 2004; Bremer and Janssen 2006; Harley 2006; Taylor et al. 2009; Couvreur et al. 2011; Stevens 2015).

The oldest known Lophopidae represent Sarebasa^+^ group, Bisma^+^ group and Apia^+^ group. This situation match well to the most parsimonious model proposed by Soulier-Perkins et al. (2007) and to statement about ‘taxonomic conservatism’ of Lophopidae in selection of the host plants as documented in Carriona^+^, Macota^+^, Bisma^+^ and Apia^+^ groups. The Sarebasa^+^ group ancestor switched to another monocot family, the Poaceae (commelinid clade; Stevens 2015, Magallón et al. 2015). Rich and widespread fossil record of fossil representatives of Sarebasa^+^ group could result from Poaceae massive diversification end expansion in the Palaeogene (Jones et al. 2014, Magallón et al. 2015). This switch appears to have been profitable, permitting an important radiation resulting in eleven recent genera and a total of fifty-six described species. Within Sarebasa^+^ group ancestors of genera *Elasmoscelis* Spinola, 1839 (21 species) and *Lophops* Spinola, 1839 (13 species) apparently extended their distributions into Africa, and may have followed the expansion of the savannah on this continent during the Miocene (around 23 Ma). The genus *Elasmoscelis* being related to Poaceae, is more oligophagous, including a few records on core eudicots (Soulier-Perkins 2007, Stevens 2015) and their diversity benefit from the ‘diet relaxation’ (Attié et al. 2008, Wang et al. 2014).

The ancestors of Arecaceae seem to spread rapidly as in late Cretaceous times they were dispersed from their area of origin to Europe, North Africa, India, North and South America (Harley 2006, Manchester et al. 2010, Futey et al. 2012, Srivastava and Srivastava 2014). Taking into consideration the known fossil record of Lophopidae, they had been dispersing coincidently with early Arecaceae.

**The distribution of Apia^+^ group**

Ancestors of Bisma^+^ group (comprising Apia^+^ clade to which newly described genus belongs) were postulated originating in the West Pacific islands arc (Soulier-Perkins 2000). Eleven of the concerned genera are found in the terranes originating from this arc. The fossils questions the postulated area of origination of Bisma+ group and its subclade, Apia+ group and the times of their origination. *Baninus thuringiorum* Szwedo et Wappler, 2006 representing Bisma+ group, was reported from the Middle Eocene Messel Maar (Szwedo and Wappler 2006); present finding in the Palaeocene of Qiangtang Basin implies earlier than formerly supposed (Soulier-Perkins 2000) time of separation of Apia+ group. These findings support the opinion on early diversification in continental part of Asia rather, and westward migrations of ancient Lophopidae in the early Palaeocene, as suggested by Szwedo and Soulier-Perkins (2010). This migration could be related to possibility of rapid dispersal due to suitable palaeogeographic and climatic conditions (DeCelles et al. 2007; Zachos et al. 2008; Zhang et al. 2008, 2011; Polissar et al. 2009; Hetzel et al. 2011; Wang et al. 2011; Dai et al. 2012). The knowledge of the dispersal events on the northern continents has greatly improved since it has been correlated with a negative Carbon Isotope (δ^13^C) Excursion – CIE linked to a short greenhouse effect called the Paleocene-Eocene Thermal Maximum – PETM (Röhl et al. 2000, Magioncalda et al. 2004, McInerney and Wing 2011). This PETM is a 150,000 years lasting abrupt global warming event that represents the warmest period of the last 65 million years, followed by the second major hyperthermal, Eocene Thermal Maximum 2 (ETM-2), that occurred approximately 53.7 million years ago (Zachos et al. 2008). Warm and uniform climatic and biotic conditions resulted in extension of geographic ranges and rapid migration of plants and animals (Smith et al. 2006, Wedmann and Makarkin 2007, Akhmetiev 2010, Lin et al. 2010, Rust et al. 2010, Grimaldi and Singh 2012), and Palaeogene Lophopidae were subject of these events as well as their Arecaceae host plants. The earliest Eocene, since the Eocene Thermal Maximum (ETM2) and the subsequent Early Eocene Climatic Optimum (Zachos et al. 2008), could be a good estimation for migration of ancient Bisma+ group lophopids to the Indian subcontinent. Such statement is supported also by analysis of Cercopoidea and Fulgoroidea biogeography presented by Liang (1998). As the Earth’s climatic belts changed dramatically during the Cenozoic, the isolated genus *Bisma* Distant, 1907 in the Ceylon appears as the relic of wider distribution in the past, according to the ‘ousted relicts’ concept (Eskov and Golovatch 1986, Eskov 1987).

Soulier-Perkins (2000) discussed the possible routes of Apia+ taxa migration. Collision of the East Philippines−Jalmaher−South Caroline Arc with the Australian Plate at the north New Guinea margin at about 25–20 Mya (Hall 2002, 2009; Hill and Hall 2002; Hall et al. 2011) enabled the first appearance of the Apia^+^ group in the New Guinea. Later, during the Pliocene-Pleistocene, the ancestors of modern *Magia* species possibly migrated to the Australia. From the middle Miocene until the Pliocene (15–5 Ma) increasing levels of aridity were influencing the habitats and vegetation in Australia (Martin 2006). However, at the beginning of the Pliocene, a brief period of warm and moist conditions occurred, which was followed by strong periods of aridity. Period that fluctuated between increasing aridity and a brief return of mesic conditions, may support dispersal and may have fractured populations among multiple, localized refugia, which resulted in increased diversification of taxa (Maekawa et al. 2003; Rowe et al. 2008; Fujita et al. 2010). Notwithstanding of these dispersal, extinction and diversification events the Bisma+ (including the Apia+ group) Lophopidae present ‘taxonomic conservatism’ in respect to their host plants selection (Soulier-Perkins et al. 2007). The modern taxa of the Apia^+^ group appears as relatively young descendants of the Palaeocene ancestors, with evolutionary history reaching mid-Cretaceous and reflected in fossil record (Szwedo and Wappler 2006, Szwedo and Soulier-Perkins 2010), distribution (Soulier-Perkins 2000) and trophic relationships (Soulier-Perkins et al. 2007).

References

Akhmetiev MA (2010) Paleocene and Eocene floristic and climatic change in Russia and Northern Kazakhstan. Bull Geosci 85(1): 77–94.

Attié M, Bourgoin T, Veslot J, Soulier-Perkins A (2008) Patterns of trophic relationships between planthoppers (Hemiptera: Fulgoromorpha) and their host plants on the Mascarene Islands. J Nat Hist 42:1591–1638 doi:10.1080/00222930802106963

Baker WJ, Couvreur TLP (2012) Biogeography and distribution patterns of Southeast Asian palms. In: Gower D, Johnson K, Richardson JE, Rosen B, Rüber L, Williams S (eds) Biotic evolution and environmental change in Southeast Asia. Cambridge University Press, Cambridge, pp 164–190

Baker WJ, Couvreur TLP (2013a) Global biogeography and diversification of palms sheds light on the evolution of tropical lineages. I. Historical biogeography. J Biogeo 40:274–285 doi:10.1111/j.1365-2699.2012.02795.x

Baker WJ, Couvreur TLP (2013b) Global biogeography and diversification of palms sheds light on the evolution of tropical lineages. II. J Biogeo 40:286–298 doi:10.1111/j.1365-2699.2012.02794.x

Bremer K, Janssen T (2006) Gondwanan origin of major monocot groups inferred from dispersal–vicariance analysis. Aliso 22:21–26

Cai XY, Fu JH (2003) Paleocene and Eocene Palaeobiocoenotic feature in the Dazhuoma section at Gangni village of Qiangtang Basin. J Northwest Univ (Nat Sci Ed) 33:443–446

Cockerell TDA (1920) Eocene insects from the Rocky Mountains. Proc US Natl Mus 57(2313): 233–260

Couvreur TLP, Forest F, Baker WJ (2011) Origin and global diversification patterns of tropical rain forests: inferences from a complete genus-level phylogeny of palms. BMC Biol 9(44):1–12 doi:10.1186/1741-7007-9-44

Dai JG, Zhao XX, Wang CS, Zhu LD, Li YL, Finn D (2012) The vast proto-Tibetan Plateau: New constraints from Paleogene Hoh Xil Basin. Gondw Res 22:434–446 doi:10.1016/j.gr.2011.08.019

DeCelles P, Quade J, Kapp P, Fan M, Dettman D, Ding L (2007) High and dry in central Tibet during the Late Oligocene. Earth Plan Sci Lett 253:389–401 doi:10.1016/j.epsl.2006.11.001

Dransfield J, Uhl NW, Asmussen CB, Baker WJ, Harley MM, Lewis CE (2008) Genera Palmarum: The evolution and classification of palms. Royal Botanic Gardens, Kew, United Kingdom, Kew Publishing, 744 pp

Eiserhardt WL, Svenning J-C, Kissling WD, Balslev H (2011) Geographical ecology of the palms (Arecaceae): determinants of diversity and distributions across spatial scales. Ann Bot 108:1391–1416 doi:10.1093/aob/mcr146.

Eskov KY, Golovatch SI (1986) On the origin of trans-Pacific disjunctions. Zool Jahrb Abt Syst Ökol Geogr Tiere 113:265–285

Eskov KY (1987) A new archaeid spider (Chelicerata: Araneae) from the Jurassic of Kazakhstan, with notes on the socalled ‘Gondwanan’ ranges of recent taxa. Neues Jahrb Geol P-A 175:81–106

Friedrich O, Norris RD, Erbacher J (2012) Evolution of middle to Late Cretaceous oceans – A55 m.y. record of Earth’s temperature and carbon cycle. Geology 40:107–110 doi:10.1130/g32701.1

Fujita MK, McGuire JA, Donnellan SC, Moritz C (2010) Diversification and persistence at the arid–monsoonal interface: Australia-wide biogeography of the Bynoe’s gecko (*Heteronotia binoei*; Gekkonidae). Evolution 64(8):2293–2314 doi:10.1111/j.1558-5646.2010.00993.x

Futey MK, Gandolfo MA, Zamaloa MC, Cúneo R, Cladera G (2012) Arecaceae fossil fruits from the Paleocene of Patagonia, Argentina. Bot Rev 78:205–234 doi: 10.1007/s12229-012-9100-9

Gomez-Navarro C, Jaramillo C, Herrera F, Wing SL, Callejas R (2009) Palms (Arecaceae) from a Paleocene rainforest of northern Colombia. Am J Bot 96(7):1300−1312 doi:10.3732/ajb.0800378

Govaerts R, Dransfield J (2005) World checklist of palms. Royal Botanic Gardens, Kew, United Kingdom, Kew Publishing, 235 pp

Grimaldi D, Singh H (2012). The extinct genus *Pareuthychaeta* in Eocene ambers (Diptera: Schizophora: Ephydroidea). Canad Entomol 144:17–28 doi:10.4039/tce.2012.5

Hall R (2002) Cenozoic geological and plate tectonic evolution of SE Asia and the SW pacific: computer based models, reconstructions and animations. J Asian Earth Sci 20:353–431 doi:10.1016/S1367-9120(01)00069-4

Hall R (2009) Southeast Asia’s changing palaeogeography. Blumea 54:148–161 doi:10.3767/000651909X475941

Hall R, Cottam MA, Wilson MEJ (2011) The SE Asian Gateway: History and Tectonics of the Australia–Asia Collision. Geol Soc London, Spec Publ 355, 381 pp

Harley MM (2006) A summary of fossil records for Arecaceae. Bot J Linn Soc 151:39–67 doi:10.1111/j.1095-8339.2006.00522.x

Harley MM, Baker WJ (2001) Pollen aperture morphology in Arecaceae: application within phylogenetic analyses, and a summary of record of palm-like pollen the fossil. Grana 40(1):45−77 doi:10.1080/00173130152591877

Harley MM, Morley RJ (1995) Ultrastructural studies of some fossil extant palm pollen, and the reconstruction of the biogeographical history of subtribes Iguanurinae and Calaminae. Rev Palaeobot Palynol 85:153–182 doi:10.1016/0034-6667(94)00133-5

Henderson A, Galeano G, Bernal R (1997) Field guide to the palms of the Americas. Princeton University Press, Princeton, New Jersey, USA, 363 pp

Hetzel R, Dunkl I, Haider V, Strobl M, von Eynatten H, Ding L, Frei D (2011) Peneplain formation in southern Tibet predates the India-Asia collision and plateau uplift. Geology 39(10):983–986 doi:10.1130/G32069.1

Hill KC, Hall R (2002) Mesozoic-Cainozoic evolution of Australia’s New Guinea margin in a West Pacific context. In: Hillis R, Müller RD (eds) Defining Australia: The Australian Plate as part of Planet Earth. Geol Soc Am Spec Pap/Geol Soc Austral Spec Publ 372:265−290 doi:10.1130/0-8137-2372-8.265

Janssen T, Bremer K (2004) The age of major monocot groups inferred from 800+ *rbc*L sequences. Bot J Linn Soc 146:385–398 doi:10.1111/j.1095-8339.2004.00345.x

Jones SS, Burke SV, Duvall MR (2014) Phylogeneomics, molecular evolution and estimated ages of lineages from deep phylogeny of Poaceae. Plant Syst Evol 300:1421–1436 doi:10.1007/s00606-013-0971-y

Liang AP (1998) Cladistic biogeography of Cercopoidea and Fulgoroidea (Insecta: Homoptera) in China and adjacent regions. Acta Zootax sinica 23(suppl.): 132–164

Lin QB, Huang DY (2006) Discovery of Paleocene Prophalangopsidae (Insecta, Orthoptera) in the Jiangtang Basin, Northern Tibet, China. Alcheringa 30(1):97–102 doi:10.1080/03115510608619346

Lin QB, Szwedo J, Huang DY, Stroiński A (2010) Weiwoboidae fam. nov. of ‘higher’ Fulgoroidea (Hemiptera: Fulgoromorpha) from the Eocene deposits of Yunnan, China. Acta Geol Sin-Engl Ed 84(4):751–755 doi:10.1111/j.1755-6724.2010.00263.x

Maekawa K, Lo N, Rose HA, Matsumoto T (2003) The evolution of soil-burrowing cockroaches (Blattaria: Blaberidae) from wood-burrowing ancestors following an invasion of the latter from Asia into Australia. Proc R Soc Lond B 270:1301–1307 doi:10.1098/rspb.2003.2359

Magallón S, Gómez-Acevedo S, Sánchez-Reyes LL, Hernández-Hernández T (2015) A metacalibrated time-tree documents the early rise of flowering plant phylogenetic diversity. New Phytol doi:10.1111/nph.13264

Magioncalda R, Dupuis C, Smith T, Steurbaut E, Gingerich PD. (2004) Paleocene-Eocene carbon isotope excursion in organic carbon and pedogenic carbonate: Direct comparison in a continental stratigraphic section. Geology 32: 553–556 doi:10.1130/G20476.1

Manchester SR, Lehman TM, Wheeler EA (2010) Fossil palms (Arecaceae, Coryphoideae) associated with juvenile herbivorous dinosaurs in the upper Cretaceous Aguja Formation, Big Bend National Park, Texas. Int J Plant Sci 171:679–689 doi:10.1086/653688

Martin HA (2006) Cenozoic climatic change and the development of the arid vegetation in Australia. J Arid Environ 66:533–563 doi:10.1016/j.jaridenv.2006.01.009

McInerney FA, Wing SL (2011) The Paleocene–Eocene thermal maximum: A perturbation of carbon cycle, climate, and biosphere with implications for the future. Annu. Rev. Earth Planet. Sci. 39, 489–516. doi: 10.1146/annurev-earth-040610-133431

Nel A, Prokop J, Nel P, Grandcolas P, Huang DY, Roques P, Guilbert E, Dostál O, Szwedo J (2012) Traits and evolution of wing venation pattern in paraneopteran insects. J Morphol 273(5):480−506 doi:10.1002/jmor.11036

Palazessi L, Barreda V (2007) Major vegetation trends in the Tertiary of Patagonia (Argentina): A qualitative paleoclimatic approach based on palynological evidence. Flora 202(4)328–337 doi:10.1016/j.flora.2006.07.006

Palmweb (2015) Palmweb: Palms of the World Online. Published on the internet http://www.palmweb.org/node/2. Accessed on 4 March 2015

Pan AD, Jacobs BD, Dransfield J, Baker WJ (2006) The fossil history of palms (Arecaceae) in Africa and new records from the Late Oligocene (28–27 Mya) of north-western Ethiopia. Bot J Linn Soc 151:69–81 doi:10.1111/j.1095-8339.2006.00523.x

Petrulevičius JF, Rust J, Wappler T, Solorzano Kraemer MM, Soulier-Perkins A (2010a) Vicariant high-latitude genus in the Lower Eocene, the case of tropical planthoppers (Fulgoromorpha: Lophopidae) in the Northern Denmark and Patagonia, Argentina. The 5^th^ FossilsX3 - The 5^th^ International Conference on Fossil Insects, The 4^th^ World Congress on Amber Inclusions, The 4^th^ International Meeting on Continental Palaeoarthropodology, August 20-25, 2010, Capital Normal University, Beijing, China. Program and Abstracts: 162.

Petrulevičius JF, Rust J, Wappler T, Solórzano Kraemer M M, Soulier Perkins A (2010b) Géneros bihemisféricos de insectos en el Eoceno inferior de Laguna del Hunco (Patagonia, Argentina) y Mo clay (Dinamarca). In: Vizcaíno SF, Ballent S, Morel EM, Bargo MS (eds) X Congreso Argentino de Paleontología y Bioestratigrafía – VII Congreso Latinoamericano de Paleontología, 20 al 24 de septiembre 2010, Universidad Nacional de La Plata, La Plata, Argentina: Facultad de Ciencias Naturales y Museo: 238 pp

Polissar PJ, Freeman KH, Rowley DB, McInerney FA, Currie BS (2009) Paleoaltimetry of the Tibetan Plateau from *D/H* ratios of lipid biomarkers. Earth Plan Sci Lett 287:64–76 doi:10.1016/j.epsl.2009.07.037

Röhl U, Bralower TJ, Norris RD, Wefer G. (2000) New chronology for the late Paleocene thermal maximum and its environmental implications. Geology 28 (10): 927–930 doi:10.1130/0091-7613(2000)28<927:NCFTLP>2.0.CO;2

Rowe KC, Reno ML, Richmond DM, Adkins RM, Steppan SJ (2008) Pliocene colonization and adaptive radiations in Australia and New Guinea (Sahul): Multilocus systematics of the old endemic rodents (Muroidea: Murinae). Mol Phylogenet Evol 47:84–101 doi:10.1016/j.ympev.2008.01.001

Rust J, Singh H, Rana RS, McCann T, Singh L, Anderson K, Sarkar N, Nascimbene PC, Stebner F, Thomas JC, Solórzano Kraemer M, Williams CJ, Engel MS, Sahni A, Grimaldi D (2010) Biogeographic and evolutionary implications of a diverse paleobiota in amber from the early Eocene of India. Proc Natl Acad Sci USA 107(43):18360–18365 doi:10.1073/pnas.1007407107

Smith T, Rose KD, Gingerich PD (2006) Rapid Asia–Europe–North America geographic dispersal of earliest Eocene primate *Teilhardina* during the Paleocene–Eocene Thermal Maximum. Proc Natl Acad Sci USA 103(30):11223–11227 doi:10.1073/pnas.0511296103

Soulier-Perkins A (2000) A phylogenetic and geotectonic scenario to explain the biogeography of the Lophopidae. Palaeogeogr Palaeoclimat Palaeoecol 160:239–254

Soulier-Perkins A (2001) The phylogeny of the Lophopidae and the impact of sexual selection and coevolutionary sexual conflict. Cladistics 17:56–78 doi:10.1111/j.1096-0031.2001.tb00111.x

Soulier-Perkins A, Ouvrard D, Attié M, Bourgoin T (2007) Evolutionary patterns in biogeography and host plant association: ‘taxonomic conservatism’ in Lophopidae (Hemiptera, Fulgoromorpha). Syst Entomol 32:305–311 doi:10.1111/j.1365-3113.2006.00365.x

Srivastava R, Srivastava G (2014) Fossil fruit of *Cocos* L. (Arecaceae) from Maastrichtian-Danian sediments of central India and its phytogeographical significance. Acta Palaeobot 54:67–75 doi:10.2478/acpa-2014-0003

Stevens PF (2015) Angiosperm Phylogeny Website. Version 12, July 2012 [updated since 2001 onwards]. http://www.mobot.org/MOBOT/research/APweb/ 23-02.2015 update

Stroiński A, Szwedo J (2012) The oldest known Lophopidae planthopper (Hemiptera: Fulgoromorpha) from the European Paleocene. Geobios 45(4):413–420 doi:http://dx.doi.org/10.1016/j.geobios.2011.10.007

Szwedo J (2011) *Ordralfabetix sirophatanis* gen. et sp. n. – the first Lophopidae from the Lowermost Eocene Oise amber, Paris Basin, France (Hemiptera: Fulgoromorpha). Zootaxa 2822:52–60

Szwedo J, Soulier-Perkins A (2010) Hopping in Palaeo-World – new proposal for migration routes of Lophopidae (Hemiptera: Fulgoromorpha). 13^th^ International Auchenorrhyncha Congress, 7^th^ International Workshop on Leafhoppers and Planthoppers of Economic Significance, 28^th^ of June - 2^nd^ of July 2010, Vaison-la-Romaine, France. Museum national d’Histoire naturelle, Paris, Département de Vaucluse: 28–29

Szwedo J, Stroiński A, Lin QB (2013) Discovery of Flatidae planthopper (Hemiptera: Fulgoromorpha) in the Palaeocene of Northern Tibet and its taxonomic and biogeographic significance. Geodiversitas, 21(4):291–298 doi:10.5252/g2013n4a

Szwedo J, Wappler T (2006) New planthoppers (Insecta: Hemiptera: Fulgoromorpha) from the Middle Eocene Messel Maar. Ann Zool 56(3):555–566

Taylor TN, Taylor EL, Krings M (2009) Paleobotany. The Biology and Evolution of Fossil Plants. 2^nd^ Edition. Academic Press, 1252 pp

Wang GC, Cao K, Zhang KX, Wang A, Liu C, Meng YN, Xu YD (2011) Spatio-temporal framework of tectonic uplift stages of the Tibetan Plateau in Cenozoic. Sci China Earth Sci 54(1):29–44 doi:10.1007/s11430-010-4110-0

Wang RR, Stroiński A, Szwedo J, Bourgoin T, Liang AP (2014) Recent dispersal and diet relaxation might explain the monotypic and endemic genus *Montrouzierana* Signoret, 1861 in New Caledonia (Hemiptera: Fulgoromorpha: Tropiduchidae). Ann Zool 64:693–708 doi:10.3161/000345414X685974

Wedmann S, Makarkin VN (2007) A new genus of Mantispidae (Insecta: Neuroptera) from the Eocene of Germany, with a review of the fossil record and palaeobiogeography of the family. Zool J Linn Soc 149: 701–716 doi:10.1111/j.1096-3642.2007.00273.x

Zachos JC, Dickens GR, Zeebe RE (2008) An early Cenozoic perspective on greenhouse warming and carbon-cycle dynamics. Nature 451:279–283 doi:10.1038/nature06588

Zhang KX, Wang GC, Cao K, Liu C, Xiang SY, Hong HL, Kou XH, Xu YD, Chen FN, Meng YN, Chen RM (2008) Cenozoic sedimentary records and geochronological constraints of differential uplift of the Qinghai-Tibet Plateau. Sci China Earth Sci 51(11):1658–1672 doi:10.1007/s11430-008-0132-2

Zhang KX, Wang GC, Ji JL, Luo MS, Kou XH, Wang YM, Xu YD, Chen FN, Chen RM, Song BW, Zhang JY, Liang YP (2011) Paleogene-Neogene stratigraphic realm and sedimentary sequence of the Qinghai-Tibet Plateau and their response to uplift of the plateau. Sci China Earth Sci 53(9):1271–1294 doi:10.1007/s11430-010-4048-2

Zhang S (2009) Geological Formation Names of China (1866–2000). Springer, 818 pp
